# FabricSpotDefect: An annotated dataset for identifying spot defects in different fabric types

**DOI:** 10.1016/j.dib.2024.111165

**Published:** 2024-11-24

**Authors:** Farzana Islam, Sumaya, Md Fahad Monir, Ashraful Islam

**Affiliations:** a Center for Computational & Data Sciences, Independent University, Bangladesh, Block B, Bashundhara R/A, Dhaka 1229, Bangladesh; b Department of Computer Science and Engineering, Independent University, Bangladesh, Block B, Bashundhara R/A, Dhaka 1229, Bangladesh

**Keywords:** Fabric defect detection, Computer vision, Data augmentation, YOLOv8 annotations, COCO format annotations, Quality control in textiles, Roboflow dataset

## Abstract

The FabricSpotDefect dataset is, to the best of our knowledge, the first dataset specifically designed to accurately challenge computer vision in detecting fabric spots. There are a total of 1014 raw images and manually annotated 3288 different categories of spots. This dataset expands to 2300 augmented images after applying six categories of augmentation techniques like flipping, rotating, shearing, saturation adjustment, brightness adjustment, and noise addition. We manually conducted annotations on original images to provide real-world essence rather than augmented images. Two versions are considered for augmented images, one is YOLOv8 resulting in 7641 annotations and another one is COCO format resulting in 7635 annotations. To reduce overfitting and to improve model robustness augmentation technique is required, which eventually increases data diversity. This dataset consists of various types of fabrics such as cotton, linen, silk, denim, patterned textiles, jacquard fabrics, and so on, and spots like stains, discolorations, oil marks, rust, blood marks, and so on. These kinds of spots are quite difficult to detect manually or in other traditional methods. The images were snapped in home lights, using basic everyday clothes, and in normal conditions, making this FabricSpotDefect dataset established in real-world applications. The dataset is organized in a way that makes it easy to use for training, testing, and validating machine learning (ML) models and can be reused at any time since this dataset is real and authentic. Researchers and Developers are free to use this prebuilt dataset to work with artificial intelligence (AI) tools that enhance quality control in the textile industry, such as checking the quality of fabrics used in clothing or medical textiles such as surgical gloves, masks, gauze and aprons and so on. The data is annotated with bounding boxes and polygons to precisely mark spot defects. This dataset is available in Roboflow with various formats like COCO and YOLOv8, which work with different ML frameworks. We strongly claim that our dataset is unique because it covers a wide range of fabrics and challenging spot defects often found in patterned and colorful prints, where spotting defects is especially difficult due to the complexity of the printed fabrics.

Specifications Table

**Table:** 

Subject	Textile Engineering, Computer Vision
Specific subject area	Spot defect detection in fabrics using computer vision techniques
Type of data	2D RGB digital images (.jpg)
Data collection	FabricSpotDefect dataset consists of 1014 raw RGB images and 3288 annotations of spot defects of the original images. These images were captured using three different smartphone cameras – the Samsung Galaxy Note20 (64 MP f/1.8 main camera with OIS and 1/1.72″ sensor size), Samsung Galaxy S20 FE (12 MP f/1.8 main camera with 1/1.76″ sensor size), and Samsung Galaxy A53 5 G, also having a similar 64 MP f/1.8 main camera rounded off by a 1/1.72″ sensor size). The images were processed and labeled using the online Roboflow platform. To expand the dataset six categories of augmentation techniques were used, leading to 2300 augmented images. Overall, there were 7641 YOLOv8 spot annotations and 7635 COCO spot annotations for spot defects across all the augmented images.
Data source location	The locations from where the fabric images were collected – - Independent University Bangladesh - City/Region: Dhaka - Country: Bangladesh
Data accessibility	Repository name: Mendeley Data Data identification number: 10.17632/6574nhzm8x.1 Direct URL to data: https://data.mendeley.com/datasets/6574nhzm8x/1
Related research article	None

## 1 Value of the Data

• The FabricSpotDefect dataset [1] is a beneficial resource for enriching computer vision for identifying various spots on fabrics. This dataset is even essential for identifying more challenging spots on patterned and colorful fabrics by creating ML models. Our dataset consists of 1014 raw images with a total of 3288 spot annotations manually and resulting (Train set: 2180, Valid set: 568, and Test set: 540), and for augmented images of version YOLOv8 there are total 2300 additional images where 7641 spot annotations and resulting (Train set:6533, Valid set: 568, and Test set: 540), and for augmented images of another version COCO there are 7635 spot annotations in total 2300 additional images and resulting (Train set:6527, Valid set: 568, and Test set: 540). This dataset is publicly available in Mendeley and classified as “spot only” making it easier for researchers to work on different AI models and can lead to innovations.
• Through image processing, FabricSpotDefect is expected to work in the textile industry to precisely denote quality, efficiency, and perfection in fabrics. This dataset concludes flawless detailed manual labeling with different categories of fabrics which makes it helpful for various fabric-related works.
• In addition, FabricSpotDefect is publicly available on the Mendeley platform which ensures easy accessibility and encourages the research community to develop new inventions regarding computer vision. Since FabricSpotDefect is the first-ever dataset that works on the intermixture of fabrics and varieties of spots, it's challenging for newcomers and AI-based textile analysis and quality control developers.
• The FabricSpotDefect works on spots that come from ink, water, oil marks, dye, chemical, rust, blood, and other irregular patches and the challenging issue comes when fabric's colorful prints and spots appear together where detecting spots is quite difficult due to similar matches. Capturing images from running videos in the industry and detecting spots instantly is one of the most difficult issues faced today. This dataset addresses this challenge by offering a dedicated resource for accurately identifying and classifying a broad range of spot defects. Despite these challenges, we have succeeded in identifying spots even on highly intricate fabrics, which are especially popular among people in the Indian subcontinent.
• This dataset provides a sufficient source of “Spot only” defects and helps researchers and developers to innovate reliable AI models for solving real-world tasks for example: fabrics used in medical items, such as bandages, aprons, gloves, and masks and exporting international clothes where quality is the priority.
• For industrial applications, human tiredness can lead to avoiding spots while working under huge pressure in factories. Employing this dataset for an automated spot detection system can be less time-consuming for industries.

## 2 Background

Automated spot detection through image processing, helps them meet these demands by improving quality, is time-consuming, and lowers operating costs [2]. AI-based models are already used to detect faults and reduce the workload for fabric inspection [3]. In modern worlds, industries like textiles use computer vision technology to improve quality to achieve better outcomes in the field of defect detection [4]. Each manufacturer has a different way of constructing a defect, and a maximum of 235 varieties of defects are found [5]. Approximately 90 % of basic defects can be found manually in fabrics, whereas complex patterned fabrics are much more challenging. Most of the fabrics may contain a minor significant flaw as well as surface flaws. The prices vary due to the fabrics, just 45–65 % price of the first quality fabrics are considered when selling the second quality fabrics [6]. Manual visual inspection methods are often affected by human factors, such as reduced concentration and leading to lower accuracy. Manual inspections rely on the experience and judgment of human experts, making it difficult to provide precise, measurable results for defect detection. According to reports from the textile industry, there is only 60 % to 75 % manual inspection is correct [7].

## 3 Data Description

**Fig. 1:**
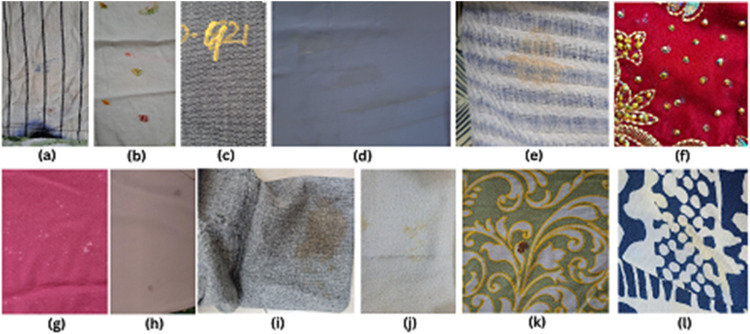
Different spot samples of raw fabric images without annotation where (a) ink stain (b) paint spot (c) marker spot (d) makeup stain (e) rust stain (f) glue spot (g)detergent stain (h) oil stain (i) coffee stain (j) food spot (k) blood spot, and (l) sweat stain.

**Fig. 2:**
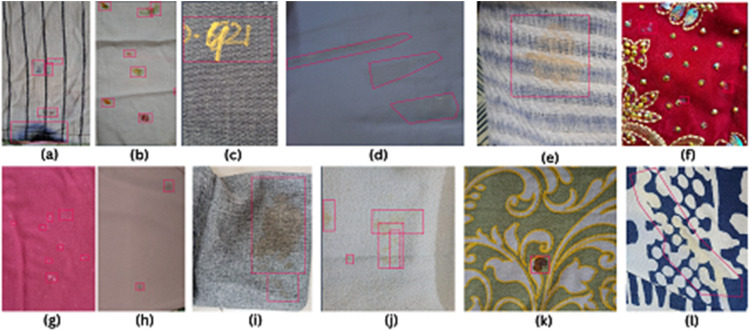
Different spot samples with annotated bounding boxes and polygon annotation in red color where (a) ink stain (b) paint spot (c) marker spot (d) makeup stain (e) rust stain (f) glue spot (g)detergent stain (h) oil stain (i) coffee stain (j) food spot (k) blood spot, and (l) sweat stain.

The FabricSpotDefect dataset contains 1014 raw images, each annotated with bounding boxes and polygon annotation using the Roboflow annotation tool [8]. The images have various spot defects, such as oil stains, dye stains, and other similar marks, all grouped under the single class “Spot”. Fig. 1 shows sample RGB images with spot defects and their corresponding annotations. Classifying all spot defects under one class, "Spot" simplifies detection, allowing ML models to focus on identifying spots regardless of their variations in appearance (Fig. 2).

To enhance the dataset and ensure there are enough examples for training ML models, six types of augmentation techniques were applied to each annotated image. This process expanded the dataset to a total of 3314 images, combining both original and augmented images. Not only does this increase the number of training examples, but it also helps the ML-based models perform better and learn more effectively [9]. Table 1 gives a summary of the FabricSpotDefect dataset. It covers what the images show, the type of files, the total count of original and modified images, information about the annotations, how the data was gathered, and where one researcher might find this dataset useful.

**Table 1:** Dataset information/description briefly.

Image Contains	Mixed fabric types, such as plain, regularly printed, and irregularly printed fabrics of varying image sizes.
**File Format**	JPG
**Number of Classes**	1 class named “Spot”.
**Total Original Images**	1014 raw images (3288 spot annotations)
**Total Augmented Images**	2300 augmented images (7641 YOLOv8 annotations and 7635 COCO annotations)
**Resolution**	All images of varying sizes were resized and made to be of equal dimensions, 416 × 416 pixels.
**Annotation Tool**	Annotations are performed using the Roboflow annotation tool, capturing a diverse range of spot defects like oil stains, dye spots, pen marks, etc.
**How Data Are Acquired**	Images were collected from daily-use home clothes, primarily under natural and household lighting to reflect real-world conditions. The images capture a variety of fabric states, including those that have undergone typical household wear and washing processes, providing realistic scenarios of spot defects.
**Use Case**	To develop an AI model that will identify spot defects in fabrics, so that the model could be used to improve the quality control process in textile production.

**Fig. 3:**
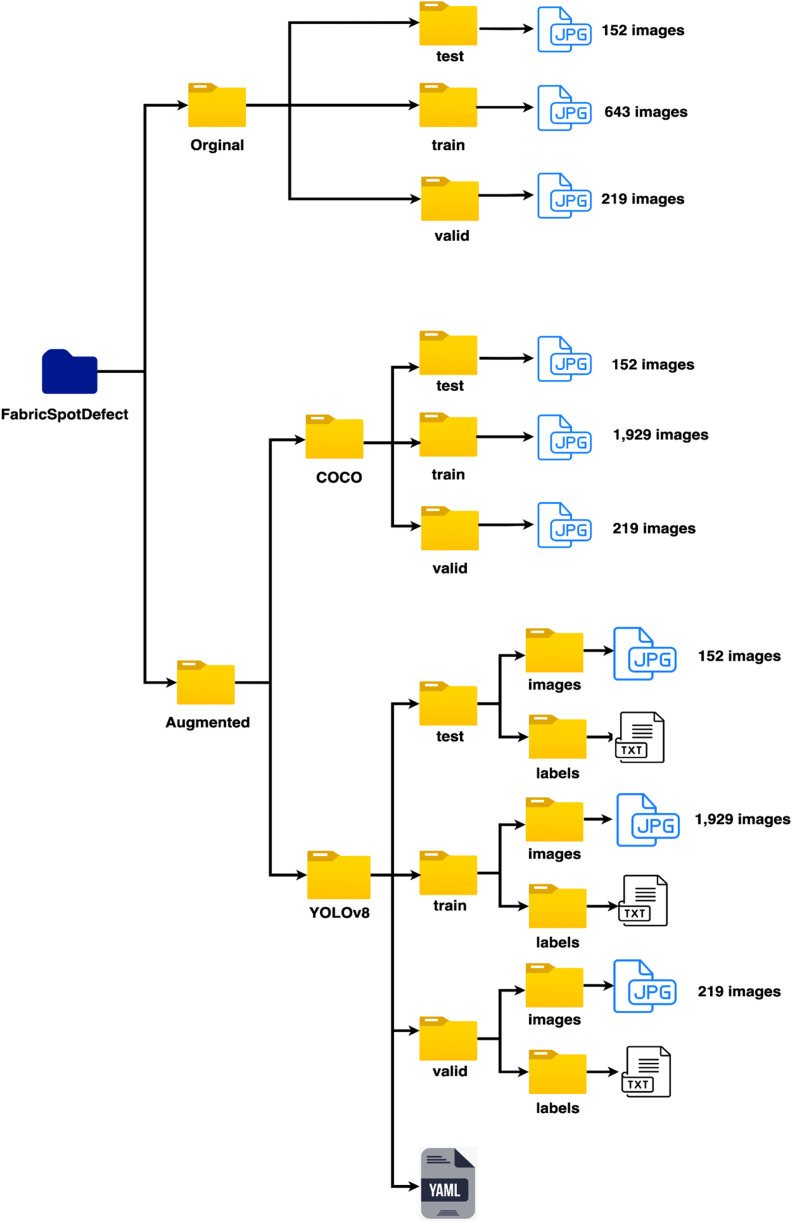
An overview of the file structure of the dataset.

In the diagram shown in Fig. 3, the FabricSpotDefect dataset is divided into two main folders: “Original” and “Augmented.” Inside the “Original” folder, there are three subfolders: “train”, “test”, and “valid.” These subfolders hold 643 images in the train folder, 152 images in the test folder, and 219 images in the valid folder. The images are in JPG format, and the labels are in COCO format. The “Augmented” folder holds images in COCO and YOLOv8 formats separated into two folders according to the format name. The COCO subfolder maintains the same folder structure as the “Original” folder, the only difference is in the number of images in the “train” folder containing 1929 images. The YOLOv8 folder is also split into “train,” “test,” and “valid,” and it has an additional file called data.yaml. This file is essential for YOLOv8 as it defines the dataset structure, including the image and label paths, class count, and class names, which are required for model training. Inside each of the “train,” “test,” and “valid” folders of YOLOv8, there are two subfolders: “images” containing the JPGs, and “labels” containing the annotations in .txt format. The FabricSpotDefect datasetʼs capability to export in both COCO and YOLOv8 formats makes it suitable for various object detection frameworks and tools, enabling easy utilization in numerous research and development projects [10].

## 4 Experimental Design, Materials and Methods

**Fig. 4:**
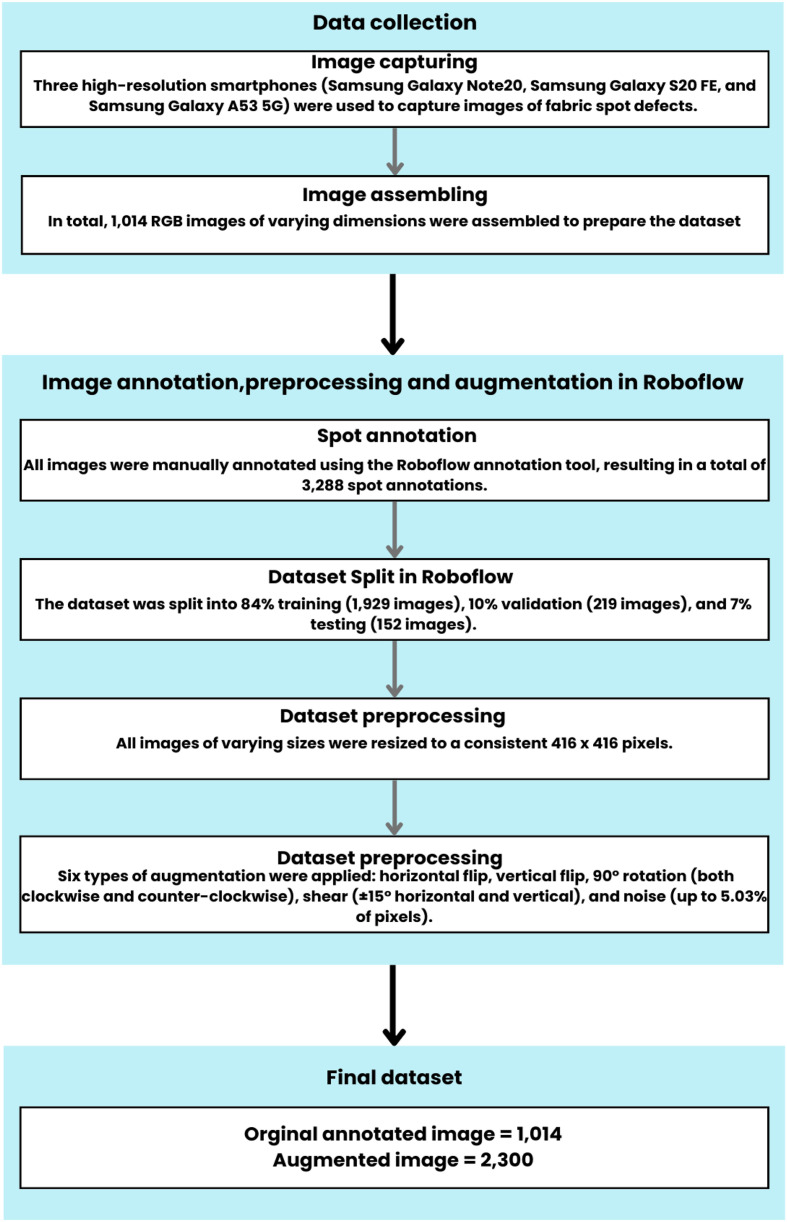
An overview of the process used to prepare the dataset.

The methodology used to prepare the FabricSpotDefect dataset is illustrated in Fig. 4 and consists of four main stages: (a) using a smartphone to capture the images of spot defects; (b) annotating the dataset using a tool named Roboflow; (c) Resizing the images with Roboflow; and (d) augmenting the data through Roboflow.

### 4.1 Image capturing

The FabricSpotDefect dataset images were captured using three different smartphones: Samsung Galaxy Note20, Samsung Galaxy S20 FE, and Samsung Galaxy A535G. They are equipped with a high-resolution camera to ensure the capture of minute details accurately. The specifications of the cameras are as follows:

• Samsung Galaxy Note20: Contains 64 MP main camera with f/1.8 aperture, optical image stabilization (OIS), 1/1.72″ sensor size.
• Samsung Galaxy S20 FE: Features a 12 MP main camera, an f/1.8 aperture, and a sensor size of 1/1.76 inches.
• Samsung Galaxy A536B: Includes a 64 MP camera with an f/1.8 aperture and a 1/1.72 sensor size.

We used the default camera settings to have consistency with all pictures and the photos were taken under normal indoor lighting, to accurately represent the natural appearance of the fabrics.

### 4.2 Spot annotation

For the FabricSpotDefect dataset, with the assistance of the Roboflow platform [8], we labeled images containing various types of fabric spot defects: stains, discoloration, oil marks, and irregular patches. The annotation of these defects is carefully done on each original image, as it plays a very significant role in developing an effective model for defect detection and classification.

Some images had polygon annotations to precisely point out the irregular or scattered spots, as shown in Fig. 1. This gives more accuracy than the bounding box and enhances the quality of training data, which will help the computer vision models to identify and classify similar defects more appropriately in new images [9]. In most of the dataset, we have used the bounding boxes as shown in Fig. 1. This effectively labels the spot defects and maintains consistency in the dataset. Bounding boxes make the annotation of spot defects fast and straightforward and cover a wide range of sizes and types of defects, hence making the dataset practical and useful for model training.

### 4.3 Dataset split in roboflow

**Fig. 5:**
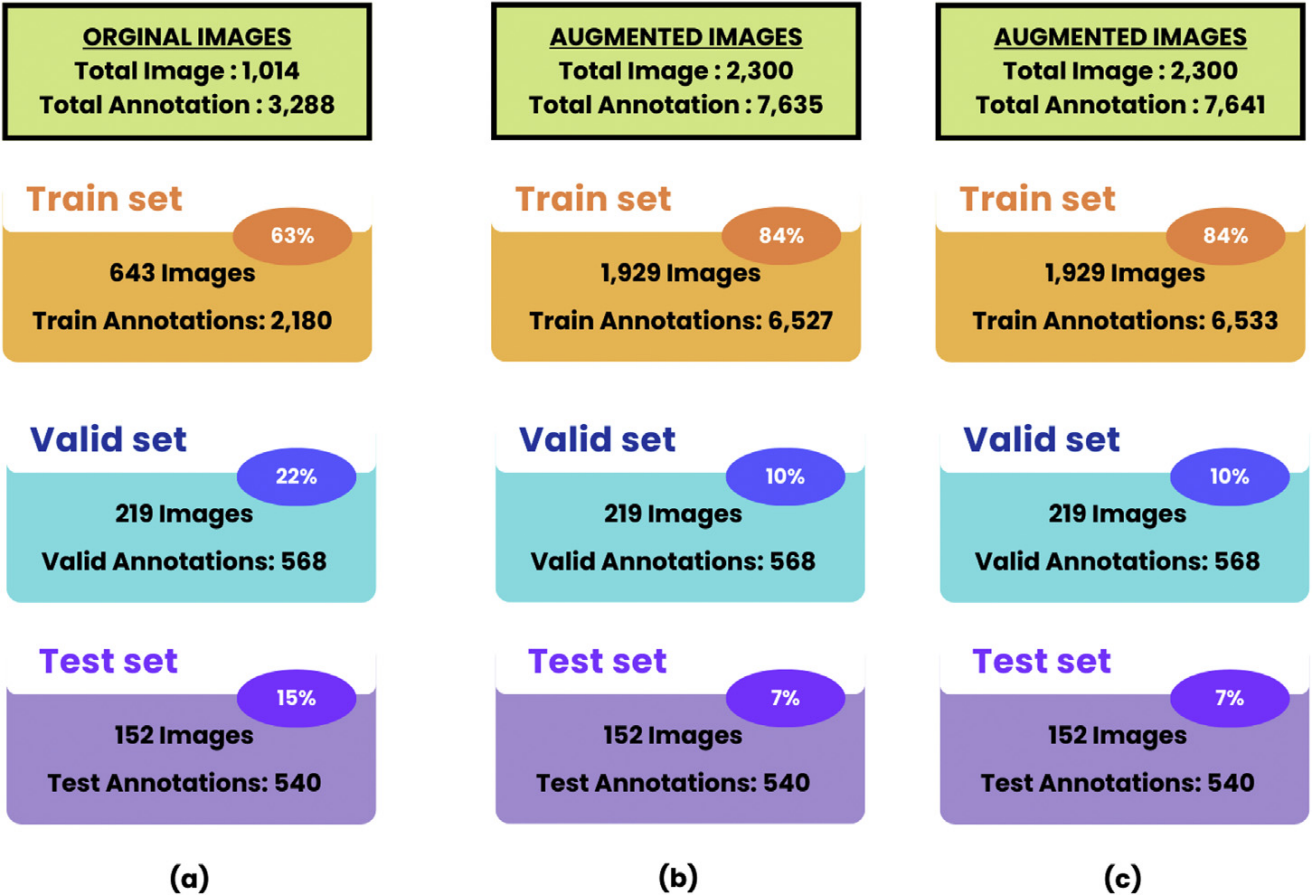
This figure shows the split of the dataset and the breakdown of the spot defect annotation. It shows (a) the split of the original images, (b) the split in the augmented images in COCO format, and (c), the split for the augmented images on YOLOv8 format.

**Fig. 6:**
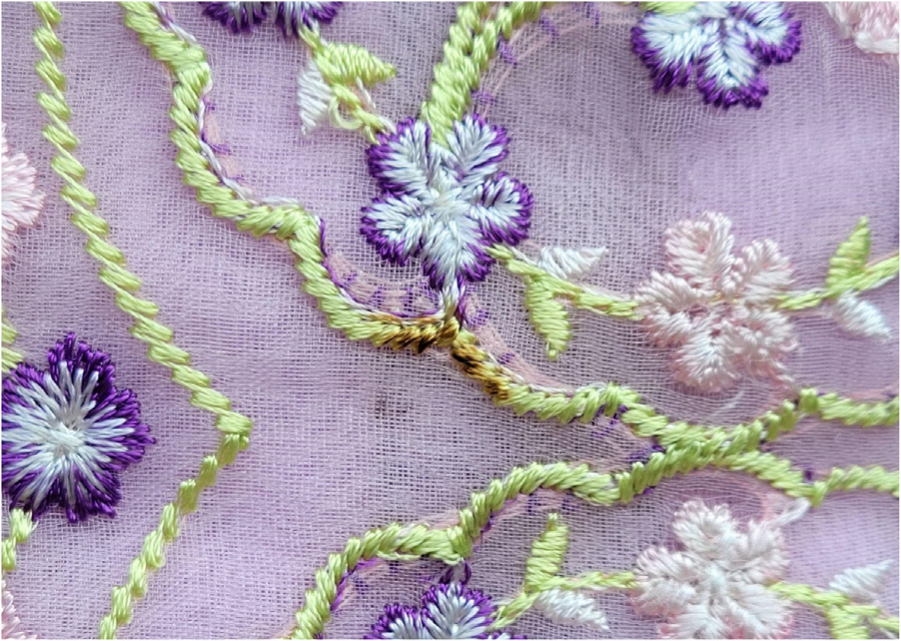
Spot image sample without augmentation.

To prepare the dataset for effective model training and evaluation, it was divided into three distinct subsets: training, validation, and testing. This division makes sure the ML models have enough data to train on, validate their performance during development, and are tested on unseen data regarding how well they perform in real-world scenarios. The original dataset includes 1014 raw images, and the breakdown of each set is clearly shown in Figs. 5 and 6.

• The training set has 643 images or 63 % of the total images. This subset has 2180 annotations that include different spot defects labeled as bounding boxes or polygons. The training set is used for the model to learn the pattern and characteristics of the spots so it can predict the spot defects effectively.
• 22 %, or 219 images, were reserved for the validation set, including 568 annotations. The validation set is useful during training because it helps in tracking performance so that some modifications can be made to avoid overfitting.
• 15 %, or 152 images, went to the testing set, containing 540 annotations. This testing subset is used for performance evaluation on previously unseen data. This testing subset is particularly important as it allows for an unbiased evaluation of the model's performance on new, unseen data, simulating real-world scenarios and helping to determine how accurately the model can identify fabric spot defects outside its training and validation experience.

Furthermore, there is an augmented dataset of 2300 synthetic images developed by various data augmentation techniques, but first, the original dataset images were again split into train, test, and valid. It is to be noted that augmentation took place after splitting the original dataset into the categories. The detailed breakdown of the dataset is provided below, as also included in Fig. 5.

• First, the training set, which includes 84 % of the images in the augmented dataset, consists of 1929 images. Next, the validation set, which represents 10 % of the images, has 219 images in total. Finally, the test set, which contains 7 % of the images, includes 152 images.
• In YOLOv8 format, there are 6533 training set annotations distributed across 1929 augmented training images. Such numerous annotations will help provide the model with several examples to learn from and help it handle different spot defect variations robustly.
• The COCO format training set consists of 6527 annotations applied to the same 1929 augmented images. The annotation count between the YOLOv8 and COCO formats differs slightly, which may be due to how each format handles the bounding boxes or annotations a little differently.
• The number of samples in the validation and test sets remains the same for both the original and augmented datasets: 568 in the validation set and 540 in the test set. This consistency makes the comparison of model performance across the different stages of training and testing very consistent.

Additionally, the dataset was divided using the Roboflow which ensures that each image is uniquely assigned to any one of the specific groups: training, validation, or test. This means that a particular image in the test set is not repeated in the validation or training sets and vice versa and hence there is no overlap. This way it made sure that the ML models would be demonstrated on completely unseen data (test sets) for a fair evaluation.

This growth in data makes the training set much richer and more diversified, hence allowing ML models to generalize well for the detection of spot defects from new unseen images. More annotations in the augmented dataset mean a model can learn different lighting conditions, orientations, and fabric patterns that allow it to be more robust and accurate in its operation.

### 4.4 Image preprocessing using Roboflow

Preprocessing is done to the dataset for effective model training, ensuring consistency to all images in the training, validation, and test sets. Using Roboflow, we applied preprocessing options like Auto-Orient and Resize. The Auto-Orient feature removes EXIF (Exchangeable Image File Format) metadata, ensuring that images are displayed uniformly across different platforms and that they appear as they are stored on disk. From the resizing feature “Stretch to” option was used to adjust all images to a consistent 416 × 416 pixels dimension [8].

### 4.5 Image augmentation using roboflow

**Fig. 7:**
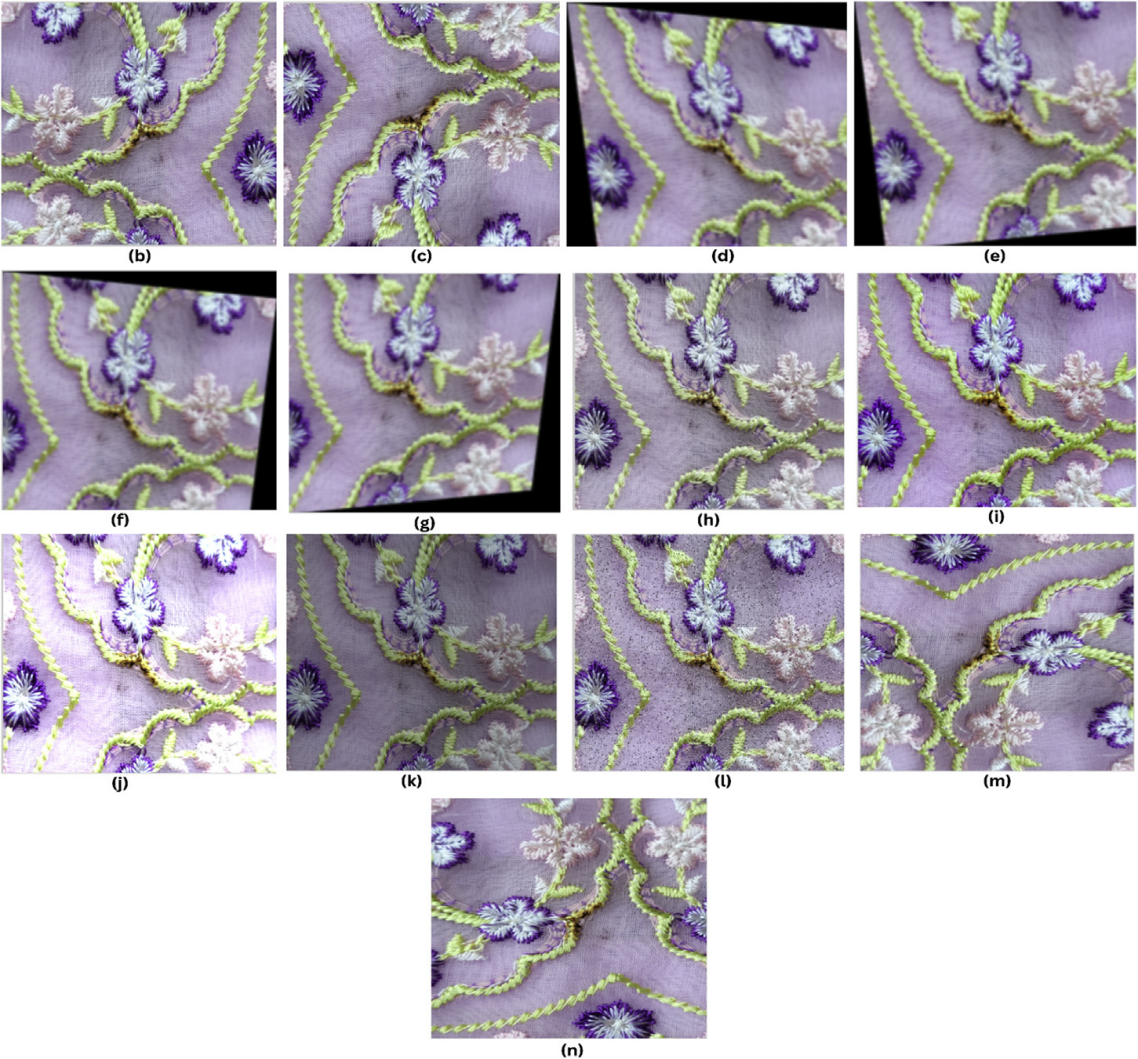
Image samples with different augmentation properties: (b) Horizontal flip, (c) Vertical flip, (d) Shear: +15°, +15°, (e) Shear: −15°, +15°, (f) Shear: +15°, −15°, (g) Shear: −15°, −15°, (h) Saturation: −25 %, (i) Saturation: +25 %, (j) Brightness: +25 %, (k) Brightness: −25 % (l) Noise: 5.03 %,(m) 90° Rotation Clockwise, (n) 90° Rotation Counter-Clockwise.

Unlike preprocessing, image augmentation is only used on the training data. We started with 1014 images (see Fig. 7 for an example before augmentation). By using different image augmentation methods like rotating, flipping horizontally and vertically, adjusting shear, saturation, brightness, and adding noise (see Fig. 7 for an example after augmentation), we generated 2300 extra images. This made our dataset bigger, with many different versions of the original images. The augmentation methods we used are:

1. *Flip (Horizontal and Vertical):* Flipping images horizontally and vertically changes the way spot defects are positioned in the image. Horizontal flips reverse the image from left to right, while vertical flips invert it from top to bottom. These transformations help the model recognize spot defects in various orientations, improving its performance on new and unseen data [9,11].
2. *90° Rotation (Clockwise and Counter-Clockwise):* Rotation does augmentation by flipping the images 90° clockwise and counter-clockwise. It gives the model the ability to identify spots and defects from different perspectives [12]. Such rotations will make sure that the model will be able to detect spot defects, whatever their alignment within the fabric is.
3. *Shear (±15° Horizontal, ±15° Vertical):* Shear Transformation involves tilting the image. Unlike rotation, where the image is turned, in shearing, one axis is kept fixed and stretches the image at a certain angle called the shearing angle. This type of stretching doesnʼt happen in rotation [13]. For our FabricSpotDefect dataset horizontal and vertical shears within *a* ± 15° range are applied. It helps to mimic situations where the fabric is not perfectly aligned with the camera. This helps the model better detect defects even when viewed from tilted angles.
4. Saturation (Between −25 % and +25 %): Saturation adjustments simulate changes in lighting, which affects the brightness and depth of colors in images. By adjusting saturation from −25 % to +25 %, the model learns to manage color changes due to different lighting or fabric types, making sure it can accurately detect defects in various settings [11,12].
5. Brightness (Between −25 % and +25 %): Brightness adjustments change how light or dark the images are, within a range of −25 % to +25 %. This helps the model work better in different lighting conditions, whether the images are taken in bright or dim settings, making it more useful in real-life situations [11].
6. Noise Addition (Up to 5.03 % of Pixels): Noise is added to up to 5.03 % of the pixels, creating random changes that mimic imperfections like camera sensor noise, fabric texture issues, or environmental factors. This method helps the model learn to tell the difference between actual defects and random disturbances, making it better at staying accurate even when the images are not perfect [11].

## Limitations and Challenges

• Despite using high-megapixel smartphones like the Samsung Galaxy Note20, S20 FE, and Samsung Galaxy A53, some images may vary in quality. This is mainly due to device-specific features like zoom in and out and so on. These factors can sometimes introduce noise or blurriness, potentially impacting the modelʼs ability to accurately detect spot defects.
• Augmentation methods included which may not fully represent all real-world situations. This might affect the model's performance when it encounters new or uncommon defect patterns that were not included in the image augmentation process.
• Images were trained and tested in natural and regular indoor lighting which might affect the model to work in industrial scenarios when light blinding situations.
• This dataset includes limited quality spot defects images and no images regarding non-defect fabrics, which leads to the model struggling to differentiate between defect and non-defect fabrics.
• The dataset contains images of stationary fabrics. This could impact the model's performance in a real production environment where fabrics are always in motion.
• The challenging part is fabrics with intricate patterns or similar shades might confuse the model performance and accuracy.

## Ethics Statement

To build this dataset no human or animal experimentation was performed. The data (images) was collected from different available fabrics.

## CRediT Author Statement

**Farzana Islam:** Conceptualization, Methodology, Data Collection, Data Curation, Formal Analysis, Investigation, Validation, Writing – Original Draft, Resources, **Sumaya:** Conceptualization, Methodology, Data Collection, Data Curation, Formal Analysis, Investigation, Validation, Writing – Original Draft, Resources, **Md Fahad Monir:** Investigation, Resources, Supervision, Writing - Review & Editing, **Ashraful Islam:** Investigation, Writing - Review & Editing, Supervision, Project Administration.

## Data Availability

Mendeley DataFabricSpotDefect: An Annotated Dataset for Identifying Spot Defects in Different Fabric Types (Original data).
